# Handbook of Historical and Geographical Phthisiology

**Published:** 1888-12

**Authors:** 


HANDBOOK OF HISTORICAL AND GEOGRAPHICAL PHTHISIOLOGY, with special reference to the distribution of Consumption in the United States. Compiled and arranged by George A. Evans, M. D., member of the Medical Society of the county of Kings. * * * New York: D. Appleton & Co. 1888.

The reader need not be alarmed at the somewhat startling name of this little volume. It is only a recently invented word corresponding to the popular and better known term, Consumption.

It begins with an interesting and exhaustive history of that disease from the time of Hippocrates down to the tubercular bacillus of Koch.

This is followed by a statement of the geographical distribution of Consumption other than the United States, and of the geographical distribution of consumption within the limits of the United States.

Large space is occupied by an attempt to give information of topography and climate of the various States and Territories and the number of deaths per thousand inhabitants which occur in each.

From the vast area of the entire country, as well as of particular States, the information upon these subjects must be vague, uncertain and unsatisfactory—though the author seems to have accomplished the utmost that he could out of the material at his disposal. The same is true of the chapter on meteorology—though the data upon this subject, while too meager, ought, as far as they go, be reliable since, they are drawn mainly from the United States Signal Service reports upon the subject of the etiology of consumption. The author discusses with care and ability such causes as temperature, humidity, dampness of soil, elevation, differences in social, hygeinic, commercial and industrial conditions, heredity, contagious transmission, individual predisposition, congenital or acquired.

All this is followed by a summary of the conclusions deduced by the author upon the curability of the disease, its treatment by residence at great altitudes and by local antiseptic treatment.

In these conclusions it is not probable that he will be followed by every professional reader, but we can heartily commend the book as a work of great interest, containing much information and many valuable suggestions.

